# Chronic Kidney Disease–Associated Pruritus and Quality of Life: Learning from Our Patients

**DOI:** 10.3390/jcm12134505

**Published:** 2023-07-05

**Authors:** Vicent Esteve-Simó, Rosa Perez-Morales, Juan Manuel Buades-Fuster, Maria Dolores Arenas Jimenez, Nuria Areste-Fosalba, Guillermo Alcalde Bezhold, Ana Blanco Santos, Emilio Sanchez Álvarez, Rafael Sanchez Villanueva, Pablo Molina, Raquel Ojeda, Mario Prieto-Velasco, Marian Goicoechea

**Affiliations:** 1 Nephrology Department, Consorci Sanitari de Terrassa, 08227 Terrassa, Spain; 2 Nephrology Department, Hospital Universitario Nuestra Señora de la Candelaria, 38010 Santa Cruz de Tenerife, Spain; rosa5ve@hotmail.com; 3 Nephrology Department, Hospital Son Llatzer, Fundació Institut d’Investigació Sanitària Illes Balears, 07120 Palma, Spain; juanm.buades@gmail.com; 4 Healthcare Management Fundación Renal Iñigo Alvarez de Toledo, 28003 Madrid, Spain; lola@olemiswebs.net; 5 Nephrology Department, Hospital Universitario Virgen de la Macarena, 41009 Sevilla, Spain; narestef@senefro.org; 6 Nephrology Department, Hospital Universitario de Araba, 01004 Vitoria, Spain; guillermo.alcaldebezhold@osakidetza.eus; 7 Fresenius Medical Care, Dialysis Center Alcobendas, Complejo Hospitalario Ruber Juan Bravo, 28006 Madrid, Spain; ana.blanco@fmc-ag.com; 8 Nephrology Department, Hospital de Cabueñes, 33394 Gijón, Spain; jesastur@gmail.com; 9 Nephrology Department, Hospital Universitario La Paz, 28046 Madrid, Spain; rafaeljesus.sanchez@salud.madrid.org; 10 Nephrology Department, Fundación para el Fomento de la Investigación Sanitaria y Biomédica (FISABIO), Hospital Universitari Dr. Peset, Universitat de València, 46017 Valencia, Spain; pablo.molina-vila@uv.es; 11 Nephrology Department, Hospital Universitario Reina Sofia, 14004 Córdoba, Spain; raqojeda@gmail.com; 12 Nephrology Department, Hospital Universitario de León, 24008 León, Spain; mprietov@gmail.com; 13 Nephrology Department, Hospital General Universitario Gregorio Marañón, 28007 Madrid, Spain; marian.goicoechea@gmail.com

**Keywords:** chronic kidney disease, haemodialysis, pruritus, opioid system, quality of life

## Abstract

Chronic kidney disease–associated pruritus is itching directly related to kidney disease that cannot be explained by any other condition. Despite technological advances in the different aspects of dialysis sessions and the best treatment for chronic kidney disease patients, it is still a common problem in our patients. The many complex physiological mechanisms involved, the different hypotheses made over the years on the aetiology of the condition, and the great clinical variability may partially explain the limited knowledge about this problem and the difficulties in treating it. The presence of all these factors leads to the persistence of unpleasant symptoms, which must affect the disease burden and quality of life of kidney patients. Through the presentation of an illustrative clinical case, the aim of this review article is to highlight the need for adequate diagnosis and an improved approach to all aspects of chronic kidney disease–associated pruritus, in view of the heavy burden of the disease and the huge impact on the patient’s quality of life.

## 1. Introduction

Chronic kidney disease–associated pruritus (CKD-aP) is itching directly related to kidney disease that cannot be explained by any other condition [1,2,3].

The many complex physiological mechanisms involved, the different hypotheses made over the years on the aetiology of the condition, and the great clinical variability may partially explain the limited knowledge about this problem and the difficulties in treating it. CKD-aP is reported to affect up to 70% of patients with end-stage kidney disease, with 37% experiencing moderate-to-severe itching [4,5,6,7].

Technological advances in the characteristics of dialysis sessions and better treatment of patients with chronic kidney disease (CKD) have helped reduce the impact of CKD-aP. However, it is still relatively common in our patients, often remaining underdiagnosed [8,9,10].

Current pruritus treatment options include a combination of topical treatments, antihistamines, gabapentinoids, µ-opioid receptor antagonists and κ-agonists, antidepressants, alterations to dialysis, and phototherapy. However, treatment responses to these therapies are variable. Furthermore, they are used off-label and are not exempt from severe adverse effects.

The presence of all these factors often means that patients and even nephrologists do not give pruritus its due importance. This can lead to the persistence of unpleasant symptoms, which must affect the disease burden and quality of life of kidney patients [9,11,12,13].

Through the presentation of an illustrative clinical case, the aim of this review article is to highlight the need for adequate diagnosis and an improved approach to all aspects of CKD-aP in view of the heavy burden of disease and the huge impact on the patient’s quality of life.

### 1.1. CKD-aP: Learning from Our Patients

An 83-year-old male patient was transferred from his satellite dialysis centre to the dialysis unit at his referral hospital because of chronic intractable pruritus.

His previous medical history included no known drug allergies, grade 1 obesity, dyslipidaemia, coronary heart disease with a severely decreased ventricular ejection fraction of ischaemic origin, revascularised (drug-eluting stent in the right coronary artery and anterior descending artery), atrial fibrillation treated with oral anticoagulants, and a complete atrioventricular block with a pacemaker implant.

The patient had been on a regular haemodialysis programme for 30 months because of CKD of unknown aetiology. Vascular access was through a left-jugular tunnelled venous catheter, which had been replaced multiple times because of severe infectious complications from *S. aureus*, without endocarditis.

The main symptom reported by the patient after starting dialysis was generalised intractable pruritus with no visible skin lesions. The intensity of the itching was 8 out of 10 points on the visual analogue scale. The patient reported the itching was unbearable and distressing, with a constant need for scratching, which made it impossible for him to sleep or rest for any reasonable period, progressively affecting his state of mind and causing frequent mood swings. He also felt more irritable with both himself and his immediate family, which was interfering with his personal and social relationships and having a big impact on his quality of life. He scored 17 out of 21 on the Itch Severity Scale.

Lab tests showed blood count and ferrokinetic values were adequate. Erythropoietic agents and intravenous iron were administered at the end of the haemodialysis (HD) sessions (haemoglobin 11 g/dL, ferritin 450 mg/dL, transferrin saturation 28%). There was no evidence of significant abnormalities in other biochemical, tumour, inflammatory, or immunological markers. The parameters related to mineral bone disorders (MBDs) showed serum calcium, phosphorus, and parathyroid hormone values (Ca 8.4 mg/dL, P 4.5 mg/dL, PTHi 350 pg/mL) within the appropriate limits recommended by the Spanish nephrology guidelines for MBD [14] while only on treatment with sevelamer carbonate. The patient received post-dilution online hemodiafiltration treatment with good haemodynamic tolerance and adequate objective parameters of dialytic efficacy (3 sessions/week, 4 h/session, HD Filter Fx80, calcium bath 2.5 meq/L, blood flow [Qb] 400 mL/min, dialysate flow [Qd] 500 mL/min, recirculation 12%, urea clearance × dialysis time [Kt] 52).

He had received specific treatment with body moisturising creams, topical corticosteroids, and even occasional intravenous corticosteroids in dialysis, without success. The dialysis filter had also been changed twice, replacing the initial modified cellulose triacetate filter with a high-flow polysulfone filter and, thereafter, with a high-permeability Helixone filter.

He had previously received intravenous antihistamines in dialysis and oral antihistamines at home (dexchlorpheniramine maleate 5 mg intravenously [IV] post-dialysis and 2 mg orally in tablets every 6 h), but they were discontinued for lack of effect and causing drowsiness. Because of the lack of response, the patient was started on pregabalin, progressively increasing to doses above those recommended in dialysis (150 mg/day). However, the pruritus persisted, and he developed several side effects, including drowsiness over most of the day and severe gastrointestinal disorders, leading to pregabalin withdrawal. He was also included in a clinical trial with tricyclic antidepressants (Tryptizol 25 mg/day at bedtime), but this was also ineffective. Finally, he was referred to an outpatient dermatology clinic, where ultraviolet B (UVB) phototherapy was indicated. He had a total of six sessions, with a temporary improvement of the itching, but it came back a few months after the treatment. As a result of the many failed treatment regimens listed above, the patient is currently being assessed for “compassionate use” medical treatment with kappa-opioid receptor agonists.

### 1.2. Diagnostic Approach to CKD-aP

The precise diagnosis of CKD-aP initially requires the exclusion of other conditions classically associated with pruritus, essentially dermatological (atopic dermatitis, psoriasis, prurigo nodularis, xerosis, scabies, or insect bites) or systemic diseases, (HIV infection or hyperthyroidism), liver disorders (primary biliary cholangitis), and oncological processes, as well as neuropathic (post-herpetic itch or brachio-radial pruritus due to spinal-nerve impingement) or psychogenic disorders (obsessive-compulsive disorder, substance abuse, delusions of parasitosis) [13,15,16,17,18]. In our case, the patient’s medical history and the absence of significant abnormalities in the biochemistry determinations carried out made it possible to reasonably rule out systemic, hepatic, or oncological causes. As he had no primary skin lesions, our patient was not initially assessed by dermatology; the development of primary skin lesions in kidney patients with pruritus would require them to be referred to dermatology for treatment. If in doubt about the origin of the lesions, dermatologists should assess whether they are, in fact, primary skin lesions, pointing to the itching being dermatological in origin or lesions secondary to scratching, which can be common to all aetiologies, including CKD-aP [7,19,20,21].

One of the main problems encountered after the diagnosis of CKD-aP is the assessment of its intensity and impact on quality of life [11,22,23,24]. Until now, the only way to assess the presence, intensity, or evolutionary course and response to the treatment of CKD-aP has been through patient-reported outcomes (PROs) [25,26].

PROs are divided into patient-reported experience measures (PREMs) and patient-reported outcomes measures (PROMs) [27,28]. PREMs, which focus on how patients feel about their experiences of health or illness, are used to garner information about satisfaction with service delivery in a clinical setting or describe the patient’s experience of a therapy or treatment plan. PROMs are questionnaires for standardised and quantitative measurement of a patient’s health status at a given time, with the intention of capturing how they feel and/or how their disease affects them, ageing, functional status, treatment, quality of life, and mental well-being. Their main clinical utility is to prescribe and monitor targeted and personalised treatments based on outcomes (referral to a psychologist, change in dialysis prescription, the administration of medication to calm itching, etc.).

There are many PROM scales available to assess the intensity of pruritus [29,30,31,32]. However, many of these scales have been used in clinical studies, not in routine clinical practice, so it is difficult to establish a scale of choice [32,33]. Classically, in the diagnosis of pruritus, these measurement scales are divided into unidimensional measures, addressing a single symptom at a time (generally intensity), and multi-dimensional measures, which assess more than one symptom (for example, duration, changes in symptoms over time, and quality of life associated with pruritus) [15,31,34].

The main PROM scales are shown in Table 1. The unidimensional scales include the visual analogue scale (VAS), the numerical rating scale (NRS), and the verbal numerical rating scale (VNRS) [35,36,37]. The VAS [35] is the most widely used scale. Initially developed to measure pain, it is a line graph representation with the left end marked as “no itching” and the right end marked as “worst itching imaginable”. Patients draw a vertical line at the point on the marked scale indicating how severe their itching is. The NRS [36] rates itch severity from 0 to 10, where 0 represents no itching and 10 represents the worst itching imaginable, and the VNRS [37] lets patients choose from four degrees of itching: no itching or mild, moderate or severe itching. They are all easy to administer and have similar reliability and validity [38].

Two of the most used multi-dimensional scales are the 5-D Itch Scale and the Itch Severity Scale (ISS) [39,40,41]. The 5-D Itch Scale [39] was designed as a brief instrument to assess the presence of pruritus over time. This scale measures the intensity, how long it has lasted, whether it is getting better or worse, its distribution, and its effect on quality of life in the last 2 weeks. It has a score ranging from 5 to 25. The Itch Severity Scale (ISS) [40] measures duration, frequency, pattern, intensity, distribution, treatment, accompanying symptoms and itching sensation, and the effect on quality of life. It has a score ranging from 0 to 21. Although widely used in different clinical trials [21,34,38], the main disadvantage of these multi-dimensional scales is that they are difficult to apply in routine clinical practice.

The self-assessed severity questionnaire, or ABC questionnaire [41], however, is simpler. It enables patients with CKD-aP to be categorised into three groups (A, B, or C) based on the severity of their pruritus and the associated symptoms. They are categorised into a single group, from group A (with CKD-aP) to group C (CKD-aP with lesions from scratching associated with itching or sleep and mood disorders). This scale is a practical instrument that is well-validated in the dialysis population and can be used routinely [38,41].

### 1.3. Impact of CKD-aP on Quality of Life

It is widely known that patients with CKD have a poorer quality of life than healthy patients in the general population [11,42,43]. Similarly, as we have seen in the case report of our patient, having CKD-aP affects the patient’s quality of life greatly, and treating the condition is, therefore, essential [22,44,45,46].

Health-related Quality of Life (HRQoL) is a vital parameter for monitoring progress and efficacy in the management of kidney disease, as well as being a fundamental variable in clinical practice and research [47,48,49]. In relation to the measurement of the quality of life in patients with CKD-aP, most of the results come from the Dialysis Outcomes and Practice Patterns Study (DOPPS) [50,51,52]. The DOPPS was an international, prospective study in HD patients, with analysis in several phases (I–VI), investigating the relationships between the outcomes obtained by the patients and the usual practices in HD, with the aim of improving clinical practice for these patients all over the world. In fact, the findings on the prevalence of CKD-aP are largely based on the answers obtained to question 20 of the Kidney Disease Quality of Life (KDQOL™) questionnaire [53], the only question related to itching, asking to what extent they have been bothered by itching in the last four weeks. This questionnaire has been validated and adapted in Spanish in the original format and its different abbreviated versions (KDQOL-36 and SF-12™) [54].

In the DOPPS I-II studies [51,52], more intense CKD-aP was associated with a lower mean score in the physical and mental components of the KDQOL test compared to patients who had only mild itching in the adjusted models (8.6 vs. 6.4 points, respectively). Patients with intense itching were also more likely to feel exhausted, a symptom which can negatively affect quality of life. The quality of life data obtained in the DOPPS studies has been corroborated in the 103 patients with CKD-aP on HD included in the ITCH study registry [41]. In that cohort, itch severity was significantly associated with poorer health-related quality of life. They also found that changes in itch intensity were associated with proportional changes in quality of life.

The dermatological involvement in CKD-aP can manifest in different ways and with wide variations in intensity, making it complicated to manage. This can all have a negative impact on the quality of life of these patients [19,55,56]. To tackle the problem, many questionnaires have been developed and validated over the last few years, mainly from dermatology, and are more sensitive and specific in detecting changes in the quality of life of patients with CKD-aP [44,56,57].

The Dermatology Quality Life Index (DLQI) and the Skindex-10 have been used in patients with CKD-aP for this purpose [58,59]. The DLQI [59], widely used and easy to administer, consists of ten questions which can be divided into six categories: symptoms–feelings, daily activities, leisure, work–school, personal relationships, and treatment. Scores range from 0 to 30. The higher the score, the worse the quality of life dermatologically. Skindex-10 [58] contains ten questions, with scores from 0 to 6, designed to assess quality of life in the last week in the domains of illness, emotional stress, and social functioning. The final score is the sum of the individual scores for all the questions. The higher the score, the worse the outcome.

As with dermatological involvement, sleep quality directly influences HRQoL in patients with CKD-aP [60,61]. There are also scales that assess the quality of sleep in relation to itching. The Itch Medical Outcomes Study (MOS) [41], developed from the Medical Outcomes Study sleep questionnaire and validated in CKD-aP, includes twelve questions that assess the effect of itching on sleep-onset latency, interruption, and daytime sleepiness.

The development of a new instrument for measuring HRQoL associated with CKD-aP is of great interest. The ItchyQoL [62,63] is an easy-to-apply questionnaire used to measure quality of life in patients with chronic itching. It can also be used to assess the response to treatment. It has been validated in most of the commonly used languages and has 22 domains (scores 1–5) on symptoms, functioning, emotions, and self-perception. In addition to the total score, a subscore can also be calculated for “symptoms”, “functioning”, and “emotions”. The higher the score, the worse the outcome.

It is important to highlight the general lack of guidelines or protocols available for the diagnosis of CKD-aP. Like other authors [34,64], we propose three simple, practical steps to properly diagnose a patient with CKD-aP. First, ask an initial screening question using question 20 of the KDQOL [53]. Second, in possible cases of CKD-aP (scores > 1 in question 20 of the KDQOL), perform an initial dermatology assessment, particularly if the patient has skin lesions, to screen for other conditions and confirm the diagnosis. Third, once the diagnosis of CKD-aP is confirmed, assess the CKD-aP further by combining any of the different approved uni- and multi-dimensional scales to establish the most appropriate treatment, and then assess response by repeating the assessments in the time frames recommended by the particular scales used.

In our case, the intensity was assessed using the horizontal VAS scale, while the severity and impact on quality of life were assessed with the ISS, so we believe these aspects were correctly assessed in the initial diagnosis. However, no protocol was followed when assessing the response to treatment for CKD-aP in our patient, as it was based on subjective data from the patient, so we need to improve on this aspect by repeating the assessment scales.

### 1.4. Impact of CKD-aP on Burden of Disease

Unlike the general population, for renal patients with advanced CKD or on dialysis, itching can recur and persist over time, leading to a heavy burden of disease and further adding to the deteriorated quality of life and resulting in poorer survival of patients with CKD-aP [45,65].

A number of previously published studies show the association between CKD-aP and a range of adverse medical conditions, the burden of disease, and the patient’s quality of life. Overall, there is a direct negative relationship between the intensity of CKD-aP and sleep disorders and depressive symptoms, worse physical condition and mental well-being, less social interaction and routine activities, increased risk of hospital admission and infections, poorer adherence to prescribed medical treatment and dialysis conditions, and higher mortality rates [6,8,11,23,61,66,67,68,69].

In our case, having CKD-aP led to greater social withdrawal and less partaking in social activities, mainly due to aesthetic problems and the feeling of itchiness, which meant he could not avoid constantly scratching in public. In addition, it would be easy to appreciate and understand from a medical perspective if our patient’s nocturnal insomnia was related to the development of other aggravating symptoms related to sleep, such as daytime sleepiness, lack of night rest, and poor quality of sleep, which could lead to him taking hypnotics, whether prescribed by his treating doctor or not. The development of depressive symptoms, such as feeling annoyed with family members and being anxious or frustrated on a personal level, would also not be surprising. These are all clinical conditions that can lead to the need for antidepressant or anxiolytic drugs, with the ensuing clinical implications, effects on daily life, and side effects that can develop over time.

There is evidence in the literature of a higher risk of hospital admission in patients with severe CKD-aP, mainly for cardiovascular causes, sepsis or bacteraemia related to the HD catheter, and severe skin infections, which then lead to increased use of intravenous antibiotics, erythropoiesis-stimulating agents, and iron supplements [7,9,68]. Most of these events were also observed in our patient, essentially due to various episodes of bacteraemia related to his vascular access for HD, leading to prolonged hospital admissions and increasing hospital costs. In our case, the patient’s depressed cardiac function and his particular characteristics meant the creation of native or prosthetic vascular access was inadvisable. However, in any event, such a move might have entailed a greater risk of lesions or skin infections resulting from the patient scratching near the vascular access site.

### 1.5. Impact of CKD-aP on Mortality Rates

The results of the initial phases of the DOPPS study (I–II) showed that patients on haemodialysis with moderate or intense pruritus had a 13% higher mortality risk in DOPPS I and 21% higher DOPPS II compared to those without pruritus, even after adjusting for numerous clinical variables and sociodemographic data [66]. However, the difference in mortality risk was attenuated after the inclusion of certain sleep variables (awake at night, sleepy during the day, and not getting enough sleep), suggesting that higher mortality rates in these patients might be explained by these CKD-aP–related sleep disturbances.

It has also been shown that the intensity of pruritus is related to a longer general recovery time after the usual HD session, a higher rate of non-adherence to treatment, and an increase in the number of HD sessions not performed [67,70,71]. These findings can also partly explain the higher mortality rates in patients with CKD-aP.

In our case, despite the fact that the patient complied with the established schedule for his HD sessions, we can easily imagine a situation where the persistence of uncontrollable pruritus can lead at some point to both non-adherence to prescribed medication and missing a regular haemodialysis session, purely out of desperation because of the huge negative impact of CKD-aP on quality of life. All these events can lead to a higher risk of life-threatening complications (such as severe hyperkalaemia or acute pulmonary oedema) and decompensation of the patient’s different comorbidities, again highlighting the vital importance of adequately treating CKD-aP.

### 1.6. Current Perspective and Future Strategies in the Therapeutic Approach to CKD-aP

The complex physiological mechanisms involved and the difficulties in diagnosis are unfortunately reflected in the lack of effective universal treatment for CKD-aP; the strategies applied by nephrologists vary greatly [43,72].

This is evident from the description of our case, in which, despite the use of the entire arsenal and all the different therapeutic alternatives, we still did not achieve adequate control of the pruritus.

Classically, an inadequate dialysis dose and the presence of biochemical abnormalities in bone-mineral metabolism, such as sustained hyperphosphataemia or severe hyperparathyroidism, were associated with the development of pruritus in patients with CKD, although these associations were not confirmed in later studies [73,74]. In our case, despite the fact that both situations were within the optimal ranges established by the MBD guidelines [14] of the nephrology society here in Spain, changes were made in the characteristics of our patient’s HD, highlighting a more traditional initial approach to this symptom.

Among the topical preparations, the treatment of skin dryness or xerosis typical of patients with CKD using moisturising creams or emollients should be a priority to reduce the itching [75,76]. Their easy application and lack of side effects make them recommendable in all cases. Other topical treatments, such as corticosteroids, widely used in skin lesions associated with pruritus, capsaicin, or pramoxine, have not shown clear, conclusive results in CKD-aP [77,78].

Antihistamines have traditionally been the most widely used therapeutic option, mainly as first-line, despite the fact that different studies show they are not effective in reducing CKD-aP [1,13,79]. The sedative effect and drowsiness are two of the typical side effects to be taken into account in this population. In the few studies carried out, mast cell stabilisers, such as zinc sulphate, montelukast, and cromoglycate, have shown positive results [80,81].

Gabapentinoid derivatives (gabapentin and pregabalin) act by inhibiting the release of neurotransmitters from the nerve terminals of presynaptic C fibres, modulating itching [82,83]. A systematic analysis that included seven studies with gabapentin found that, in six of these studies, they were able to reduce the intensity of the pruritus [84]. The lack of studies in populations with the clinical characteristics of dialysis patients and long-term studies, combined with the common occurrence of side effects (neurological symptoms, falls, fractures, and gastrointestinal symptoms), mean gabapentinoid derivatives have to be used with much care and caution. Patients intolerant to gabapentin can be treated with pregabalin, with a better pharmacokinetic and pharmacodynamic profile [79,83].

Phototherapy using ultraviolet A (UVA) light has not shown positive results in the treatment of CKD-aP. In contrast, UVB radiation has been shown to be effective in published studies and in most treated patients [78,85]. The main drawback is the development of skin cancer associated with the long duration of treatment and the high rates of recurrence after discontinuing therapy [86].

In our case, over the entire time, virtually every medical treatment available to date was used. Despite the limited evidence on most of them, it is worth noting that they were all used based on personal experience to improve our patient’s aggravating and uncomfortable symptoms and in the absence of any available treatment proven effective. Because of the lack of sufficient knowledge on managing other topical treatments, such as capsaicin, or other mast-cell inflammatory-response stabilisers, such as montelukast or cromoglycate, we did not try these options [77,79,80,81]. In our experience, gabapentin was inadvisable, considering the known serious neurological side effects and the increased risk of falls and fractures, so instead, we opted for pregabalin. Similarly, the limited use and experience in nephrology in the management of the major opioids [87] dissuaded us from using this class of drug. Perhaps the use of a serotonin reuptake inhibitor antidepressant [75,88] might have provided some improvement in the patient’s symptoms, but the long history and long list of previous treatment failures made it difficult to envisage any meaningful improvement. The clinical characteristics of our case, the considerable increase in the number of hospital visits, and the expected long duration of the phototherapy led to the patient abandoning the treatment and, thus, limited its possible beneficial effects.

As mentioned previously, the pathophysiological mechanisms involved in CKD-aP are multiple, complex, and mostly unknown [72,89]. The main mechanisms discussed in CKD-aP are summarised in Table 2. There are four main hypotheses: uraemic toxin deposition, dysregulation of the opioid system, immune system dysfunction, and peripheral neuropathy [90,91,92].

Uraemic toxin accumulation and deposition classically have been related to CKD-aP since increasing dialysis efficiency and reducing serum calcium, PTH, or phosphorous all alleviate itching in a subset of patients. Immune system dysregulation still remains a potential modulator of CKD-aP, as increased levels of eosinophils, mast cells, histamine, and tryptase have all been reported [89,90]. It is recognised that inflammation plays a key role in sensitising the small nerve fibres in the skin that carry the itching sensation to the brain, producing the uncomfortable symptom of itching. Furthermore, high levels of markers of systemic inflammation are observed in patients with CKD-aP, including high levels of T cells, white blood cells, C-reactive protein, interleukins -6 and -2, and ferritin, alongside low levels of albumin [90,91,92]. Peripheral neuropathy has been demonstrated to cause itching when diseased neurons are activated independently in the presence of pruritogens, with peripheral neuropathy highly prevalent in dialysis patients [89,91].

Currently, dysregulation of the opioid system is perhaps the hypothesis that carries the most weight. It is postulated that CKD involves an imbalance between the mu (μ) and kappa (κ) opioid receptors, which are mutually antagonistic, with an imbalance in favour of the μ receptors [92]. Therefore, pruritus is increased via μ-receptor activation or κ-receptor blockade and decreased via κ-receptor activation or μ-receptor blockade. Pruritus is a common adverse effect after administration of μ agonists and can be mediated by the modulation of serotonergic transmission and activation of the dorsal horn and itch centre in the central nervous system.

The focus of the emerging novel therapies is on the search for adequate regulation of the opioid system. There are currently several drugs with well-designed studies already carried out with satisfactory results. Nalfurafine is a peripheral kappa receptor agonist indicated for the treatment of refractory CKD-aP, capable of reducing the intensity of pruritus in haemodialysis patients in Japan [93]. However, its use is not approved in Europe because of a lack of consistency in the results obtained for improvement compared to placebo in the primary endpoint in the European population. Nalbuphine [94], a mu-opioid receptor antagonist and kappa-opioid receptor agonist, has improved pruritus in HD patients with no difference in serious adverse events between groups, although no long-term efficacy data are available. Lastly, difelikefalin [95] is a specific and highly selective peripheral kappa-opioid receptor agonist with promising significant results in the reduction of symptoms and in quality of life in CKD-aP patients. No central symptoms were observed, such as euphoria, dysphoria, or hallucinations, and there were no cases of physical dependence on opioids after its use, although various mild gastrointestinal adverse effects were somewhat more common in patients who received difelikefalin. In our patient, in view of the promising results obtained in clinical trials, we assessed the possibility of medical treatment with difelikefalin under compassionate use, now approved by the United States Food and Drug Administration (FDA) and the European Medicines Agency (EMA) for use in CKD-aP, although it is not yet available in Europe [96,97].

Difelikefalin appears relatively safe and shows promising results. Once it has marketing authorisation, its use can be considered in patients with moderate-to-severe CKD-aP. As it will be the only drug indicated for CKD-aP, once the dialysis characteristics and MBD control are adjusted and all patients apply topical treatment with emollients or moisturising cream, it will likely become the treatment of choice.

## 2. Conclusions

CKD-aP continues to be common in our patients but is very often underdiagnosed. The authors have attempted to demonstrate the need for adequate diagnosis of CKD-aP and a suitable approach to this condition because of the CKD-aP significant negative effect on the burden of disease, quality of life, and patient mortality rates. The use of validated and approved PROMs is vital in the correct assessment and management of patients with CKD-aP, so they need to be incorporated into routine clinical practice. A better understanding of the multiple pathophysiological mechanisms involved in CKD-aP needs to be combined with the latest advances in research into novel pharmacological therapies to create meaningful strategies to reduce the impact of CKD-aP on the quality of life of these patients.

## Figures and Tables

**Table 1:** Main patient-reported outcome measures (PROM) scales available for the evaluation of pruritus intensity. Visual analogue scale (VAS); numerical rating scale (NRS); verbal numerical rating scale (VNRS); 5-D Itch Scale; Itch Severity Scale (ISS); self-assessed severity questionnaire or ABC questionnaire (self-assessed disease severity score [SADS])*;* Dermatology Life Quality Index (DLQI); Skindex-10; Itch Quality of Life Scale (ItchyQoL).

Itch Rating Scales	Main Features
Unidimensional scales	
VAS	Line graph representation; Left end “no itching” and right end “worst itching imaginable”, where patients draw a vertical line at the point on the marked scale indicating how severe their itching is.
NRS	Numerical scale; Itch severity is scored from 0 to 10, where 0 represents no itching and 10 represents the worst itching imaginable.
VNRS	Verbal rating scale; Patients can choose between four grades of itching: no itching or mild, moderate or severe itching.
Multidimensional scales	
5-D Itch Scale	Divided into five dimensions with a score ranging from 5 to 25; To assess the presence of pruritus over time; It measures the intensity, how long it has lasted, whether it is getting better or worse, its distribution, and its effect on quality of life in the last 2 weeks.
ISS	Measures 10 dimensions with a score ranging from 0 to 21; It measures duration, frequency, pattern, intensity, distribution, treatment, accompanying symptoms and itching sensation, and its effect on quality of life.
ABC	Categorises the patient into three groups (A, B, or C) based on severity and symptoms; They are categorised into a single group, from group A (without CKD-aP) to group C (with lesions from scratching associated with itching or sleep and mood disorders).
DLQI	Ten questions with score ranges from 0 to 30, in which the higher the score, the worse the quality of life dermatologically; Six categories: symptoms–feelings, daily activities, leisure, work–school, personal relationships, and treatment.
Skindex-10	Ten questions with scores of 0 to 6, where the higher the score, the worse the quality of life dermatologically; Assesses quality of life in the last week in the domains of illness, emotional stress, and social functioning.
ItchyQoL	22 items (scores 1–5) on symptoms, functioning, emotions, and self-perception, where the higher the score, the worse the outcome; It measures quality of life and the response to treatment in patients with chronic pruritus.

**Table 2:** Summary of the main mechanisms discussed in CKD-aP.

Main CKD-aP Hypothesis	Theoretical Mechanisms and Pathways Involved in CKD-aP
Skin disorders	Skin atrophy Xerosis Microangiopathy
Uraemic toxin deposition	Reduced dialysis dose Increased calcium deposition on the skin Increased calcium, phosphate or PTH levels Lower iron serum levels and haemoglobin levels
Peripheral and central nervous system	Increased neuropathy susceptibility Imbalance of the opioid system: μ-opioid overexpression and κ-opioid downregulation Genetic variants of opioid receptors
Immune system dysfunction	Systemic inflammation Increased proinflammatory cytokines (CRP, IL-2, IL-4, IL-13, IL-31) Th1/Th2 lymphocyte dysregulation Inflammatory response to dialysis filters Local inflammation Increased mast cell density and activity

## Data Availability

Data are unavailable due to privacy or ethical restrictions.
